# PD24 Innovation In Chronic Obstructive Pulmonary Disease Care: Cost Effectiveness Of A New Self-Management Maintenance Program In The United Kingdom

**DOI:** 10.1017/S0266462324002885

**Published:** 2025-01-07

**Authors:** Anil Gumber, Amir J Khan, Matthew Richardson, Annabel Hong, Claire Nolan, Khaled Alqahtani, William Man, Sally Singh, Freda Smart, Chris Hedger, Linzy Houchen-Woolloff, Ala Szczepura

## Abstract

**Introduction:**

Innovation is needed for the growing number of patients with chronic obstructive pulmonary disease (COPD). Pulmonary rehabilitation (PR) is effective in improving exercise tolerance and quality of life, but these benefits do not appear to be sustained. This highlights the need for cost effective methods to maintain benefits on completion of therapy. The findings of a large trial from the UK are reported.

**Methods:**

A two-center randomized controlled trial of patients discharged from PR compared the costs and benefits of PR maintenance with standard care. National Health Service (NHS) resource use, personal expenditure, and societal costs were recorded over one year, and bottom-up costing was undertaken for the PR maintenance program. Changes in health-related quality of life were recorded using the EQ-5D-5L, and differences were compared with the level identified as significant for COPD. A cost utility analysis was undertaken from an NHS perspective; uncertainties in cost and outcome data were incorporated into a sensitivity analysis. Cost-effectiveness ratios and cost-effectiveness acceptability curves (CEACs) were computed.

**Results:**

The study included 116 patients who had finished PR within the last four weeks. The economic analysis showed that mean healthcare costs per patient for PR maintenance were approximately GBP139.72 (EUR165.57) lower than for usual care. The observed 0.118 advantage in mean quality-adjusted life-years (QALYs) (p<0.05) was above the threshold (0.051) for COPD significance. CEACs indicated there was a 97 percent chance of achieving GBP20,000 (EUR23,699.80) per QALY (NICE acceptance level ≤GBP30,000 (EUR35,549.70). Patient and societal costs increased this percentage. It was estimated that if patients with COPD completed a maintenance program following PR, the NHS could save up to GBP28.6 million (EUR33.89 million).

**Conclusions:**

Our findings confirm that a structured PR maintenance program is highly cost effective in extending the benefits of short-term PR. The trial, undertaken during COVID, also signals the potential for emerging digital innovations to provide future transformative change in delivering self-management programs to sustain health and reduce NHS costs for people living with chronic conditions.

